# The Essential Role of Selenoproteins in the Resolution of *Citrobacter rodentium*-Induced Intestinal Inflammation

**DOI:** 10.3389/fnut.2020.00096

**Published:** 2020-07-08

**Authors:** Shaneice K. Nettleford, Luming Zhao, Fenghua Qian, Morgan Herold, Brooke Arner, Dhimant Desai, Shantu Amin, Na Xiong, Vishal Singh, Bradley A. Carlson, K. Sandeep Prabhu

**Affiliations:** ^1^Department of Veterinary and Biomedical Sciences, Center for Molecular Immunology and Infectious Disease, The Pennsylvania State University, State College, PA, United States; ^2^Department of Pharmacology, Organic Synthesis Core Laboratory, Penn State Cancer Institute, Penn State College of Medicine, Hershey, PA, United States; ^3^Department of Microbiology, Immunology & Molecular Genetics, Long School of Medicine, University of Texas Health San Antonio, San Antonio, TX, United States; ^4^Department of Nutritional Sciences, The Pennsylvania State University, State College, PA, United States; ^5^Molecular Biology of Selenium Section, Mouse Genetics Program, National Cancer Institute, National Institutes of Health, Bethesda, MD, United States

**Keywords:** selenium, cyclopentenone prostaglandins, metabolism, lamina propria, enteric infection

## Abstract

Enteropathogenic *Escherichia coli* (EPEC) leads to adverse colonic inflammation associated with poor resolution of inflammation and loss of epithelial integrity. Micronutrient trace element selenium (Se) is incorporated into selenoproteins as the 21st amino acid, selenocysteine (Sec). Previous studies have shown that such an incorporation of Sec into the selenoproteome is key for the anti-inflammatory functions of Se in macrophages and other immune cells. An intriguing mechanism underlying the anti-inflammatory and pro-resolving effects of Se stems from the ability of selenoproteins to skew arachidonic acid metabolism from pro-inflammatory mediators, prostaglandin E_2_ (PGE_2_) toward anti-inflammatory mediators derived from PGD_2_, such as 15-deoxy-Δ^12, 14^- prostaglandin J_2_ (15d-PGJ_2_), via eicosanoid class switching of bioactive lipids. The impact of Se and such an eicosanoid-class switching mechanism was tested in an enteric infection model of gut inflammation by *C. rodentium*, a murine equivalent of EPEC. C57BL/6 mice deficient in Se (Se-D) experienced higher mortality when compared to those on Se adequate (0.08 ppm Se) and Se supplemented (0.4 ppm Se) diets following infection. Decreased survival was associated with decreased group 3 innate lymphoid cells (ILC3s) and T helper 17 (Th17) cells in colonic lamina propria of Se-D mice along with deceased expression of epithelial barrier protein Zo-1. Inhibition of metabolic inactivation of PGE_2_ by 15-prostaglandin dehydrogenase blocked the Se-dependent increase in ILC3 and Th17 cells in addition to reducing epithelial barrier integrity, as seen by increased systemic levels of FITC-dextran following oral administration; while 15d-PGJ_2_ administration in Se-D mice alleviated the effects by increasing ILC3 and Th17 cells. Mice lacking selenoproteins in monocyte/macrophages via the conditional deletion of the tRNA^[Sec]^ showed increased mortality post infection. Our studies indicate a crucial role for dietary Se in the protection against inflammation following enteric infection via immune mechanisms involving epithelial barrier integrity.

## Introduction

Gastrointestinal (GI) pathogens, such as enteropathogenic *Escherichia coli* (EPEC), remain a global concern (1). EPEC leads to infantile persistent diarrhea with a high mortality rate (1–3). In fact, the prevalence of EPEC in children ranges from 5 to 20% in developing countries (2). When ingested, the bacteria reside in the intestine, where they affect the epithelial barrier. One identifying feature of these enteric infections is the formation of attaching and effacing (A/E) lesions in the colon (1, 4). Enteric infections are characterized by inflammation of the GI tract somewhat similar to that seen in Crohn's disease, ulcerative colitis, dysbiosis, and colon tumorigenesis (5–9). *Citrobacter rodentium* (*C. rodentium*) is the murine equivalent of EPEC that shares approximately two-thirds of its genome with EPEC, including the bacterial pathogenicity genes, and forms A/E lesions (1, 4, 10, 11). Additionally, the immune response that arises from its colonization of the gut also mimics that of EPEC, which is associated with exacerbated inflammation as a result of the production of inflammatory cytokines such as, tumor necrosis factor (TNFα), interferon gamma (IFNγ), and interleukin (IL-6) (12). Thus, *C. rodentium* is often used as a model for EPEC (1).

Selenium (Se) is an essential trace element that functions through its incorporation as the 21st amino acid, selenocysteine (Sec), in selenoproteins. Dietary Se is incorporated as Sec into proteins via the decoding of UGA codon by tRNA^[Sec]^, which is encoded by *Trsp* (13). As part of the anti-inflammatory and redox-gatekeeper role of Se and selenoproteins, innate immune cells, such as macrophages, exhibited decreased expression of pro-inflammatory mediators in response to inflammatory stimuli only when selenoproteins were expressed (14–16). Most importantly, in inflamed macrophages that were Se replete, eicosanoid class switching led to the skewing of arachidonic acid (ARA) metabolism to increase the levels of hematopoietic-PGD_2_ synthase (H-Pgds) dependent prostaglandin D_2_ (PGD_2_) and its downstream metabolites such as Δ^12^-prostaglandin J_2_ (Δ^12^-PGJ_2_) and 15-deoxy-delta-12,14-prostaglandin J_2_ (15d-PGJ_2_) that are endowed with anti-inflammatory properties (15). As a result, the levels of prostaglandin E_2_ (PGE_2_), a product of microsomal prostaglandin E synthase-1 (Mpges-1), and other pro-inflammatory eicosanoids, such as thromboxane A_2_, were decreased (15). Inhibition of H-Pgds activity with HQL-79 or the oxidation of PGE_2_ by 15-hydroxy prostaglandin dehydrogenase (15-Pgdh) by CAY10397 led to reversal of the anti-inflammatory effects of Se (14, 17). Previous studies using the dextran sodium sulfate (DSS) model of GI inflammation further demonstrated increased PGD_2_ and its cyclopentenone metabolites in Se supplemented mice that were associated with decreased colitogenic symptoms when compared to the mice fed diets that were either deficient or adequate in Se (17). Se supplementation decreased the expression of pro-inflammatory cytokines and mediators but increased the expression of Arg-1 suggesting an increase in resolution (17). To our knowledge, epidemiological studies correlating the levels of Se to prostaglandin metabolism during enteric bacterial infections, such as EPEC, do not exist. Interestingly, there are a number of studies that report a correlation between inflammatory bowel disease (IBD), which can also develop as a result of enteric infections, where Se levels are reduced in patients with two forms of IBD, ulcerative colitis and Crohn's disease (18–20). Studies have further reported an increase in PGE_2_ in ulcerative colitis patients, while other studies have demonstrated a decrease in 15-Pgdh, a tumor suppressor, in both ulcerative colitis and Crohn's disease patients (21, 22). Higher levels of PGD_2_ have also been observed in Crohn's disease patients who experienced long-term remission (>4 years) (23). 15d-PGJ_2_ has been suggested to serve as a therapeutic agent in IBD through its role as a ligand of peroxisome proliferator activated receptor (PPARγ) (24). However, it is not clear how 15-Pgdh and 15d-PGJ_2_ are protective, particularly in GI inflammation seen during EPEC infections.

Both the innate and adaptive immune mechanisms are paramount to the clearance of *C. rodentium*. Following infection, neutrophils, monocytes, macrophages, and dendritic cells are recruited to the colonic mucosa (1). They respond to cytokines and other factors, and also result in the differentiation of the adaptive component of the immune system. For instance, group 3 innate lymphoid cells (ILC3s) and T helper 17 (Th17) cells that produce IL-17 and IL-22, are key mediators in eliminating the infection (1). In fact, lack of IL-17A and IL-17F exacerbates *C. rodentium* infection (1). More importantly, both IL-17 and IL-22 are crucial for repairing the epithelial barrier following injury, where these cytokines act on mucosal epithelial cells to induce cell proliferation and wound healing, as well as induce antimicrobial peptide production (1, 25, 26). The intestinal epithelium is continuously exposed to luminal antigens, microbiota, and various toxins, resulting in the need to form a protective barrier. In addition to the effector cytokines, IL-22, and IL-17, the integrity of the epithelium is also maintained by tight junction proteins, such as occludin, claudin, and zona occludens 1, 2, and 3 (ZO-1, 2,3), which regulate the permeability of the epithelium (27). Disruption of the intestinal epithelial barrier is a hallmark of both EPEC and *C. rodentium* infections that result in systemic bacterial dissemination (1, 9, 28). Therefore, healing of the disrupted gut epithelial barrier, following infection is a key factor that contributes to resolution.

While there are numerous reports on the regulation of immune responses during an enteric infection, there are limited studies that report the effect of Se and selenoproteins on immune responses and function following infection (29), and the effect of selenoproteins on epithelial barrier integrity following injury (30). Our studies are based on the hypothesis that Se and selenoproteins alleviate inflammation and infection via mechanisms that involve modulation of ILC3 and Th17 cells where shunting of ARA metabolism to favor the biosynthesis of anti-inflammatory mediators occupies a central role in the maintenance of the epithelial barrier integrity. Here we report that deficiency in Se is detrimental to survival, as Se-D mice succumbed to *C. rodentium* infection. Mice maintained on a Se supplemented diet elicited ILC3 and Th17 responses and expressed higher levels of *Zo-1* following infection. Furthermore, pharmacological inhibition of 15-Pgdh activity with CAY10397 decreased their ability to maintain their epithelial barrier integrity, while administration of 15d-PGJ_2_ increased the ability of mice to maintain the epithelial barrier. Mice that lacked expression of selenoproteins in monocyte/macrophages corroborated the increased mortality observed in Se-D mice lending credence to the idea that the responses require monocyte/macrophage selenoproteins where bioactive lipid mediators and enzymes, 15d-PGJ_2_ and 15-Pgdh, respectively, play a critical role. In summary, our studies show that selenoproteins mediate the effects of dietary Se, to serve as preventative and/or adjuvant therapy to efficiently resolve GI inflammation associated with an enteric bacterial infection.

## Materials and Methods

### Mice

Three-week old C57BL/6 mice (Taconic) were fed AIN-76 based semi-purified diets that only differed in the amount of Se. The mice were on Se-deficient (Se-D; <0.01 ppm Se), Se-adequate (Se-A; 0.08 ppm Se as sodium selenite), or Se-supplemented (Se-S; 0.4 ppm Se as sodium selenite) diets to ensure equilibration of Se levels as described previously (14, 17). C57BL/6 mice used in the CAY10397 and 15d-PGJ_2_ studies were placed on corresponding diets for 4 and 8 weeks, respectively. *Trsp*^*fl*/*fl*^*LysM*^*Cre*^ mice, which lack the ability to express selenoproteins in monocytes and macrophages, were generated as described earlier (31, 32). These mice were placed on Se-D or Se-A diets, along with their sex, age, and diet matched WT controls. All procedures conducted were preapproved by the Institutional Animal Care and Use Committee at The Pennsylvania State University, University Park, PA.

### *C. rodentium* Infection

Individually housed C57BL/6 and *Trsp*^*fl*/*fl*^*LysM*^*Cre*^ mice were infected with 5 × 10^9^ colony forming units (CFUs) of *C. rodentium* strain ICC 169 (nalidixic acid resistant), a gift from Dr. Margherita Cantorna, Penn State University. Infected mice were weighed, and fecal pellets were collected and plated on MacConkey agar containing nalidixic acid every two to three days, starting at day two post infection (PI). Mice were euthanized and the colonic tissue was collected for further analysis.

### Treatment of Mice With CAY10397 or 15d-PGJ_2_

CAY10397, a selective inhibitor of 15-Pgdh, was synthesized as previously described and dissolved in 3.3% v/v cell culture grade DMSO and 0.1M sodium carbonate following dilution with PBS (17). One day prior to infection, C57BL/6 mice on Se-A diet were administered 75 mg/kg of CAY10397 via oral gavage, a concentration that was effective in inhibiting 15-Pgdh activity in tissues by ~65% (17). These mice were gavaged every alternate day until day 21 PI. To examine the role of 15d-PGJ_2_ in Se-D mice, 15d-PGJ_2_ (Cayman Chemicals) was used. 15d-PGJ_2_ was evaporated under a stream of nitrogen and dissolved in PBS. One day prior to infection, C57BL/6 mice on Se-D diet were treated with 0.05 mg/kg of 15d-PGJ_2_ via intraperitoneal injection. The mice were treated daily until they cleared the infection.

### Isolation of Colonic Lamina Propria Cells

Colons were removed from mice and the colonic lamina propria (LP) cells were isolated and processed as previously described with some modifications (33). Briefly, fecal pellets, fat, connective tissue, and mucus lining of the intestine were removed from the colon. The colonic samples were cut into 5 mm pieces followed by incubation in Hank's buffered salt solution (supplemented with 0.25 M EDTA, 1M HEPES, 10% FBS and 1% Penicillin/Streptomycin) for 30 min, at 37°C with shaking. The tissues were then cut into smaller pieces and digested with collagenase type I (1 mg/ml, Worthington) and DNase I (10 ug/ml) in RPMI media containing 5% FBS, for an hour, at 37°C. A Percoll gradient of 40–80% was used to isolate the LP cells, which were recovered at the interface of the gradient following centrifugation at 800 *g* for 20 min.

### Flow Cytometry

Prior to staining with primary antibodies, cells were incubated in RPMI media containing 10% FBS, 50 ng/mL PMA, 1 μg/mL ionomycin and brefeldin A (1:1000; Biolegend) for 4 h at 37°C. Following staining with primary antibodies in PBS containing 3% FBS, cells were fixed using 4% paraformaldehyde, permeabilized, and stained overnight in permeabilization buffer. The primary antibodies were diluted as described: CD45-A700 (1:100; Biolegend, Clone: 30-F11), CD4-FITC (1:100; Biolegend, Clone: GK15), CD3-BV421 (1:100; Biolegend, Clone: 17A2), Lin-biotin conjugated (1:50; Miltenyi Biotec: cocktail containing antibodies against CD5, CD11b, CD45R, anti-7-4, anti-Gr-1 and anti-Ter119), Streptavidin-PE-CF594 (1:500; BD Biosciences), IL-17A-PE-Cy7 (1:1000; eBioscience, Clone: eBio17B7), and IL-22-APC (1:100; eBioscience, Clone: IL22JOP). Th17 cells were categorized as CD45^+^CD3^+^CD4^+^IL-17A^+^, while ILC3s were categorized as CD45^+^Lin^−^CD3^−^IL-17A^+^IL-22^+^. The gating strategy is described in detail in Supplementary Figure 1.

### ELISA

*C. rodentium* was cultured overnight in Luria Broth at 37°C. For the analysis of *C. rodentium* specific antibody responses, 100 μl of a stock concentration containing 2 × 10^9^ CFU/ml in 0.1M sodium carbonate was used to coat 96 well ELISA plates overnight at 4°C. The plates were washed three times with PBS containing 0.05% Tween-20 (wash buffer) and blocked with 200 μl of PBS containing 1% (w/v) bovine serum albumin (BSA) for 1 hour at 37°C. After blocking, the plates were washed three times with wash buffer and incubated with 100μl of diluted serum (1:50) for 90 min at 37°C. Following incubation, the plates were washed three times with washing buffer and incubated with 100 μl of biotinylated anti-mouse IgG and IgM (diluted 1:5000, Jackson ImmunoResearch) for 1 h at 37°C. The plates were incubated with 100 μl of streptavidin conjugated HRP (diluted 1:5000, Jackson ImmunoResearch) for 20 min and developed with ABTS (2,2′-azino-bis(3-ethylbenzothiazoline-6-sulfonic acid; Southern Biotech) substrate and the absorbance was recorded at 405 nm in a plate reader.

### FITC Dextran Assay

The FITC-dextran assay was performed as described (34). Briefly, prior to administration of FITC dextran, *C. rodentium* infected mice treated with CAY10397 or vehicle-control, were deprived of food and water for 4 h. PBS-diluted FITC-dextran (FD4; 500 mg/kg; Sigma Aldrich) was administered to mice via oral gavage. Mice were euthanized after 4 h, serum was collected by cardiac puncture, and centrifuged at 10,000 *g* for 10 min. Serum levels of FITC-dextran were measured in a plate fluorimeter (Synergy HTX) and the concentrations were determined using a FITC dextran standard calibration curve. Samples with hemolysis were excluded.

### Real Time PCR

RNA was isolated from colon samples using TRI Reagent (Sigma Aldrich). Taqman probes (ThermoFisher Scientific) were used to examine the expression of Cox2, Hpgds, and Mpges-1 (normalized to Gapdh) by quantitative real-time PCR. SYBR primers were used to examine the expression of Zo-1 (forward primer: 5′-TGCAGACCCAGCAAAGGT-3′; Reverse primer: 5′-GGTTTTGTCTCATCATTTCTTCAG-3′) and occludin (forward primer: 5′-GTCCGTGAGGCCTTTTGA-3′; reverse primer: 5′-GGTGCATAATGATTGGGTTTG-3′), GAPDH (forward primer: 5′-TGACATCAAGAAGGTGGTGAAGC-3′; Reverse primer: 5′-CCCTGTTGCTGTAGCCGTATTC-3′). The data is expressed as 2^−ΔΔ*Ct*^.

### Western Immunoblot

Colon (proximal) samples were homogenized in mammalian protein extraction reagent (MPER; ThermoFisher Scientific) and centrifuged at 10,000 *g* for 20 min at 4°C. Expression of candidate proteins in the supernatant were analyzed using western immunoblotting following separation of proteins by SDS-PAGE on a 6 or 12 % gel under denaturing conditions. The membranes were incubated in primary antibody to the following proteins: 15-Pgdh (1:1000, Abcam), Cox-2 (1:500, Abcam), or Zo-1 (1:500, Proteintech) overnight at 4°C. Expression of β-actin (1:20,000; Fitzgerald) or Vinculin (1:5,000; Cell Proteintech) were used as loading controls. The blots were incubated with appropriate HRP-conjugated secondary antibody for 1 hour at room temperature and were developed and imaged with West Pico chemiluminescence reagent (ThermoFisher Scientific) and G:BOX Chemi XX6 (Syngene), respectively. Densitometric evaluation of the bands was performed using Image J software (National Institutes of Health).

### Immunohistochemistry

The distal portion of the colons from mice maintained on custom diets as well as those treated with CAY10397 or 15d-PGJ_2_ and their respective vehicle controls were stored in 10% (v/v) buffered formalin. The sections were prepared at the Histopathology Core Facility, Animal Diagnostic Laboratory at Penn State University, University Park, PA. To detect Zo-1, sections were deparaffinized, followed by antigen-retrieval, and blocking with 5% goat serum. The sections were incubated in rabbit anti-Zo-1 antibody (1:500; Abcam) overnight at 4°C. The sections were washed in TBS and 0.5% hydrogen peroxide used to block any endogenous peroxidase. The sections were incubated in goat anti-rabbit secondary antibody (Vector Laboratories) for 1 h followed by incubation in ABC reagent (Vector Laboratories) for 45 min at room temperature. DAB substrate kit (Vector Laboratories) was used to develop peroxidase activity and the sections were dehydrated and mounted in dibutylphthalate polystyrene xylene (Sigma). The slides were imaged using an OLYMPUS DP74 microscope and OLYMPUS CellSens Standard software at magnification 10x. The length of well-oriented crypts of the distal colon of individual mice was also measured using the OLYMPUS CellSens Standard software.

### Data Analysis

All data were analyzed using GraphPad Prism (ver 8.0) and are reported as mean ± SEM. To calculate statistical significance, either a two-tailed unpaired *T* test, one way ANOVA, or a two way ANOVA was used. A *P* value <0.05 was considered significant. Experiments were designed and samples sizes were utilized with adequate power based on previous data.

## Results

### Selenium Deficiency Results in Decreased Survival Following an Enteric Infection

**Figure 1:**
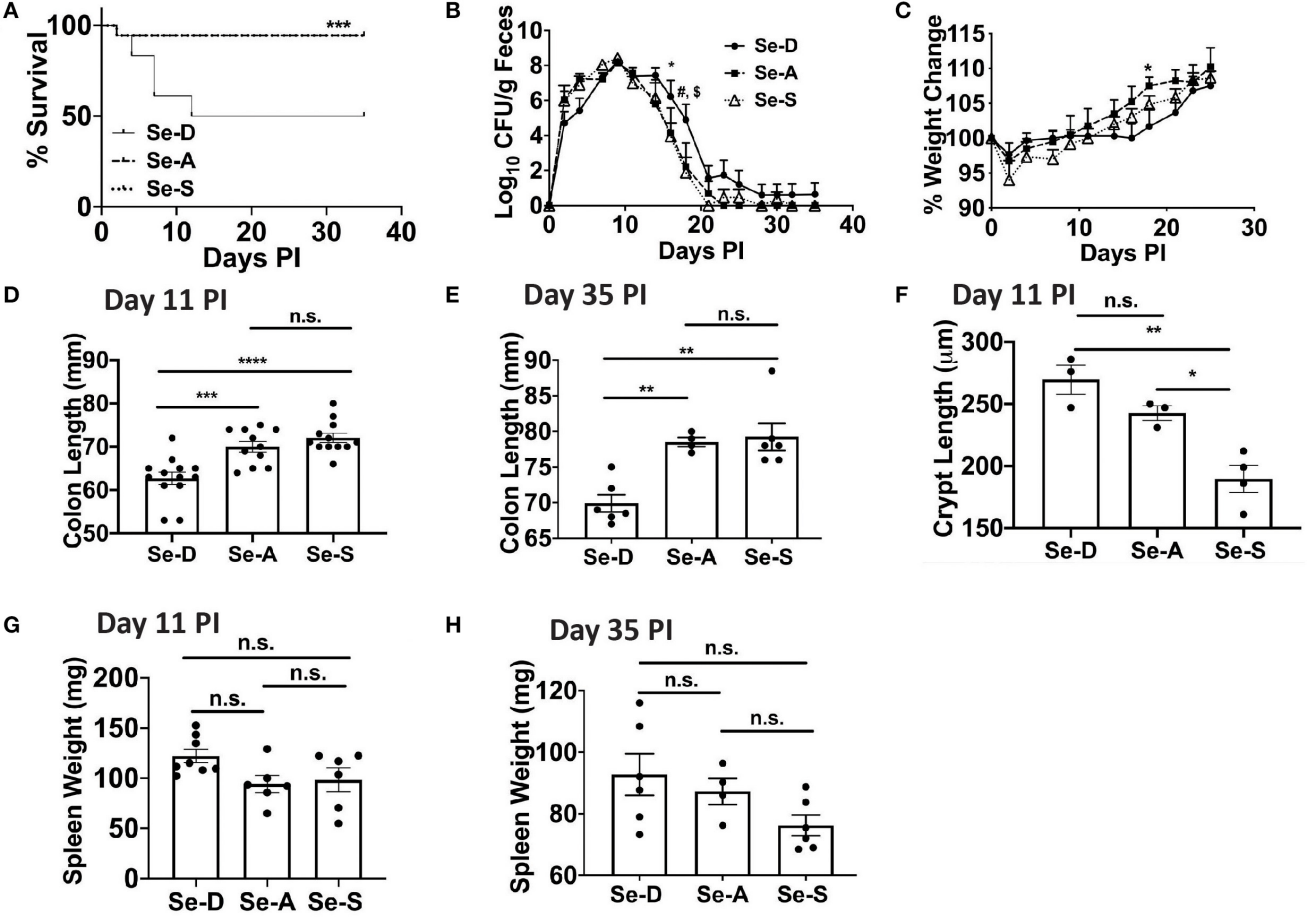
Se deficiency exacerbates enteric infection by *C. rodentium*. **(A)** Survival curve of C57BL/6 mice on Se-D, Se-A, and Se-S diets upon infection by oral gavage with 5 x 10^9^ CFU of *C. rodentium*. **(B)** Shedding curve of C57BL/6 mice on Se-D, Se-A and Se-S diets (*comparing Se-D vs. Se-A, and Se-D vs. Se-S at day 16 PI; ^#^indicates *comparing Se-D vs. Se-A at day 18 PI, ^*$*^indicates ***comparing Se-D vs. Se-S at day 18 PI). **(C)** Percent weight change of C57BL/6 mice on Se-D, Se-A and Se-S diets. **(D)** Colon length of mice on Se-D, Se-A, and Se-S diets at day 11 PI. **(E)** Colon length of mice on Se-D, Se-A, and Se-S diets at day 35 PI. **(F)** Colonic crypt length of mice on Se-D, Se-A, and Se-S diets at day 11 PI. **(G)** Spleen weight of Se-D, Se-A, Se-S mice at day 11 PI. **(H)** Spleen weights of Se-D, Se-A, Se-S mice at day 35 PI. Data indicate mean ± SEM of two independent experiments and *n* = 3–13 mice/diet group, two-way ANOVA **(B, C)**, one-way ANOVA **(D–H)**. *P < 0.05, **P < 0.01, ***P < 0.001, ****P < 0.0001, n.s., not significant.

To elucidate the effects of Se/selenoproteins on the clearance of the enteric bacteria, C57BL/6 mice were placed on Se-D, Se-A, and Se-S diets for 8 weeks and were infected with *C. rodentium* by oral gavage. Enumeration of bacterial burden in the feces was used as an indicator of severity of infection and the ability of mice to resolve the infection in the colon. We hypothesized that Se-A and Se-S mice would experience a lower bacterial burden and greater survival when compared to the Se-D mice as a result of an increased ability to mount a better immune response and increased epithelial barrier integrity. The survival curve revealed the most striking data, where 50% of the Se-D mice succumbed to infection by the peak of infection compared to the 94% of mice on Se-A and Se-S diets that survived post day 11 following infection (Figure 1A). We observed that the mice experienced a peak of infection between days 7 and 11 and started clearance of the bacteria at around day 14 PI and were completely clear by day 35 (Figure 1B). Interestingly, the only difference in the shedding curve was observed on days 16 and 18 during the later phase of infection when the mice were clearing the bacteria. Se-A and Se-S mice displayed lower bacterial burden when compared to Se-D mice on days 16 and 18 (Figure 1B). Similarly, differences in body weight amongst the groups were observed during the clearance of the bacteria, on day 18 PI, where Se-D mice experienced a greater weight loss compared to the mice on Se-A or Se-S diets (Figure 1C). While differences were observed at days 16 and 18, most of the data were analyzed on day 11 PI, as this represented the peak of infection. Shorter colons were seen in Se-D mice at day 7 (not shown) and 11 PI, which persisted even after these mice were able to clear the infection (day 35) (Figures 1D,E). Furthermore, Se-S mice had shorter colonic crypt length compared to Se-D and Se-A mice at day 11, suggesting that Se-D and Se-A mice experienced a more severe colonic hyperplasia (Figure 1F). Comparison of splenic weight, a parameter that indicates disease severity and a generalized inflammatory response, revealed a trend for lighter spleens in Se-A and Se-S mice at days 11 PI and 35 PI (Figures 1G,H), though the differences were not significant. Taken together, these findings suggest that Se supplementation at adequate to supplemental levels is crucial for combating the pathophysiology of infection, while inefficient and lack of timely resolution of inflammation was observed in Se-D mice.

### Mice on Se-A and Se-S Diets Express a Higher Percentage of ILC3s and Th17 Cells and Display Greater Zo-1 Expression

**Figure 2:**
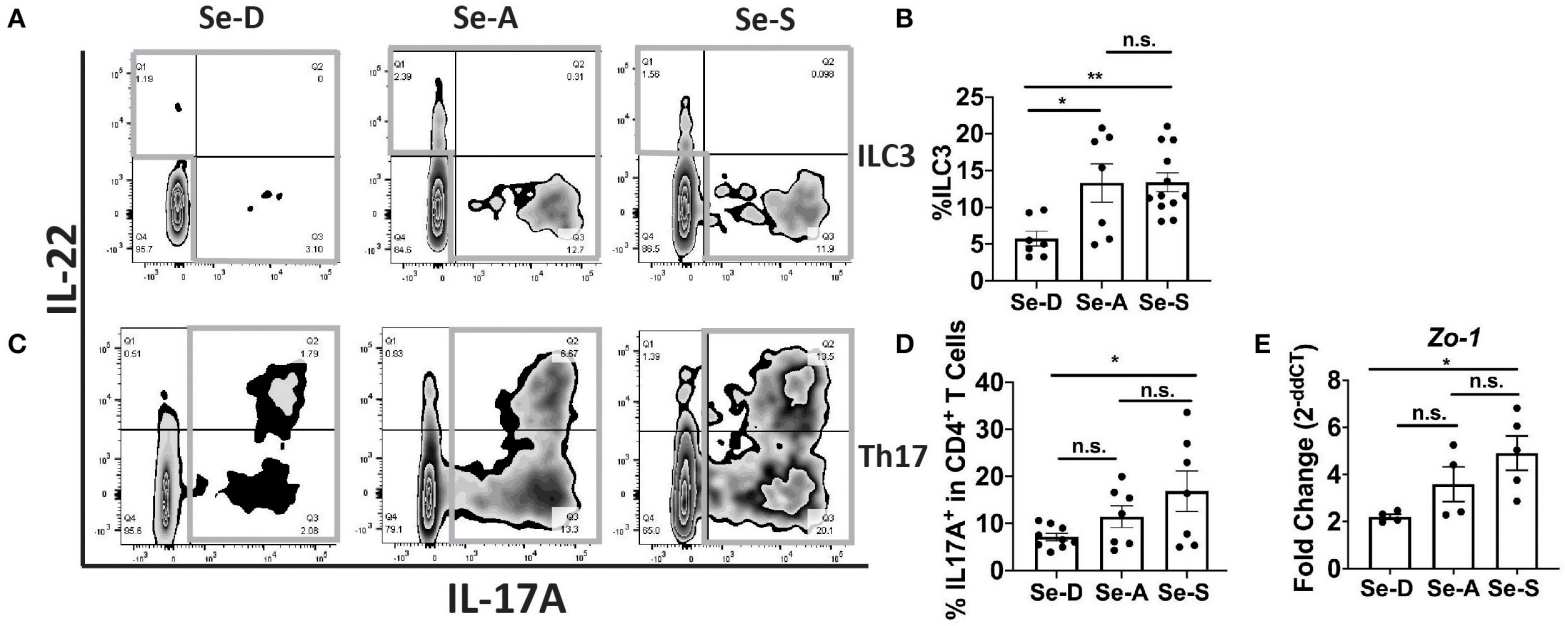
Effect of dietary Se on the ILC3 and Th17 cells PI indicates Se-A and Se-S mice have greater levels of these cells. **(A)** Representative flow plot and **(B)** quantification of ILC3s isolated from the cLP at day 11 PI. Gray box around representative flow plots indicate IL-17A^+^IL-22^+^ cells. **(C)** Representative flow plot and **(D)** quantification of IL-17A^+^ cells of CD4^+^ T cells indicating Th17 cells isolated from the cLP at day 11 PI. Gray box around representative flow plots indicate IL-17A^+^ cells. **(E)** *Zo-1* mRNA expression in colon of mice on Se-D, Se-A, and Se-S diets at day 11 PI. Data indicate mean ± SEM of two to three independent experiments and *n* = 4–12 mice/group; one-way ANOVA **(B,D,E)** *P < 0.05, **P < 0.01, n.s., not significant.

Flow cytometric analysis of ILC3 and Th17 cells from the colonic LP revealed a greater percentage of ILC3 cells (CD45^+^Lin^−^CD3^−^IL-17A^+^IL-22^+^) in the Se-A and Se-S mice compared to the mice on Se-D diet, at day 11 PI (Figures 2A,B). There was also a greater number of ILC3s per one million CD45^+^ cells in Se-A and Se-S mice compared to Se-D mice (Supplementary Figure 2A). Similarly, a higher percentage of Th17 cells (CD45^+^CD4^+^CD3^+^IL-17A^+^) was observed in the Se-S mice when compared to Se-D mice, at day 11 PI (Figures 2C,D). Furthermore, a higher number of Th17 cells per one million CD45^+^ cells in Se-S mice compared to Se-D mice were also observed (Supplementary Figure 2B). In addition to ILC3s and Th17 responses, humoral responses also provide protection during the infection, as mice lacking B cells and IgG experience exacerbated infection (35). We measured the serum anti-*C. rodentium* IgG and IgM levels of Se-D, Se-A, and Se-S mice, where we observed no differences at day 11 PI (Supplementary Figures 2C,D). We further examined if Se status had an impact on the barrier integrity of the colon after *C. rodentium* infection given the modulation in ILC3 and Th17 cells. Realtime PCR analysis revealed a 2.7-fold higher expression of *Zo-1* in Se-S mice when compared to the colons of Se-D mice (Figure 2E). We also observed higher transcript levels of *Zo-1* in Se-S mice compared to Se-D mice at day 16 PI, when the mice were in the process of clearing the bacteria (Supplementary Figure 3A). In addition to *Zo-1*, the Se-S mice showed an increase trend in *occludin* at day 9 PI, the start of the peak of infection, and also during the clearing phase of day 16 PI, compared to Se-D mice (Supplementary Figures 3B,C). Furthermore, immunohistochemistry (IHC) staining of colonic sections for Zo-1 revealed that the distal colons of Se-S mice also had greater expression of the protein compared to the Se-D mice (Supplementary Figure 3D). Together, these results suggest that Se supplementation increases ILC3 and Th17 cells that are required for clearance of the enteric pathogen as well as maintaining the epithelial barrier integrity of the colon during infection.

### Inhibition of 15-Pgdh Exacerbates Infection in Se-A Mice

**Figure 3:**
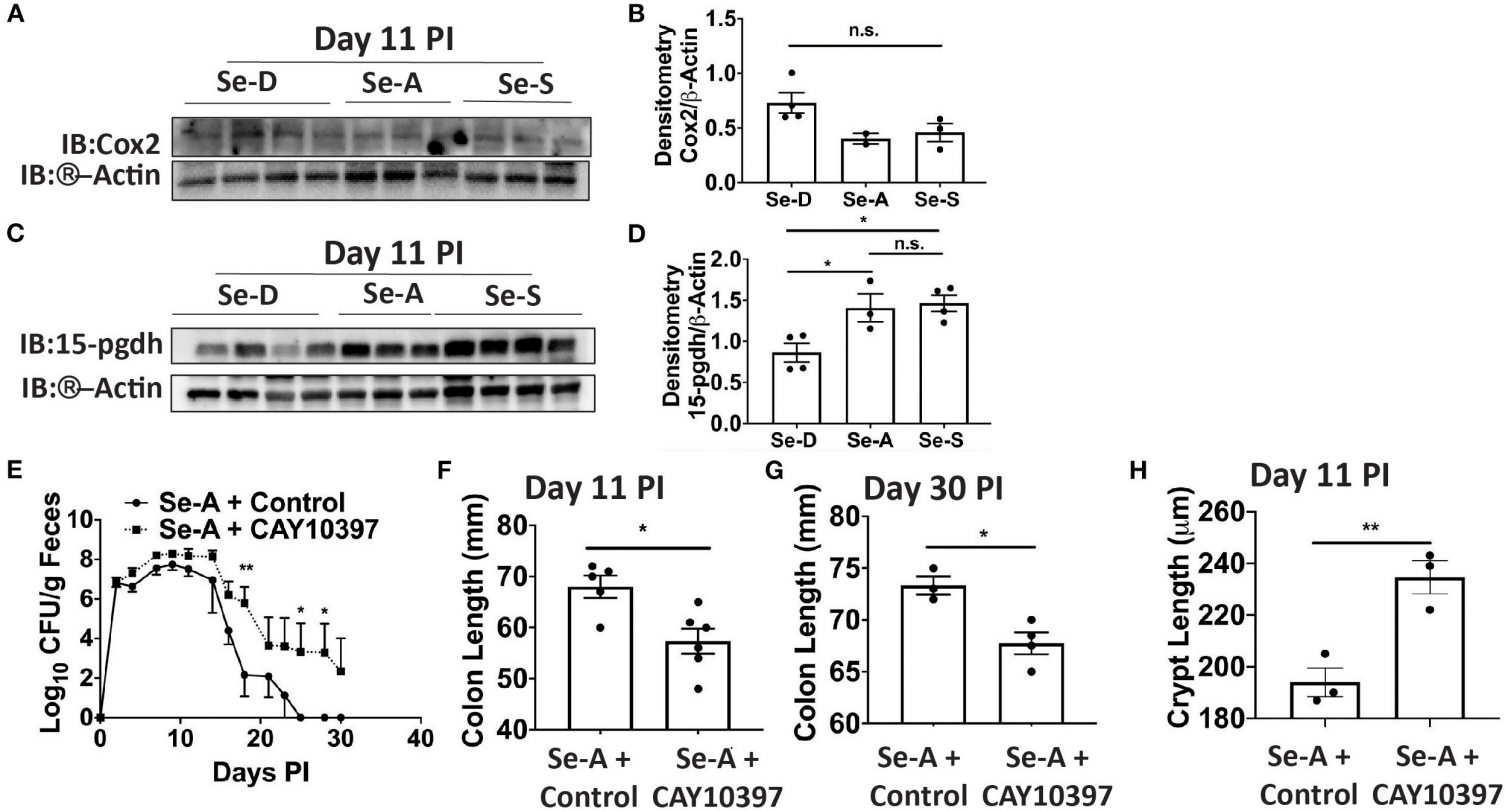
Inhibition of 15-Pgdh activity in Se-A mice exacerbates infection. **(A)** Representative western immunoblot and **(B)** quantification of Cox2 in the colonic extracts of Se-D, Se-A, and Se-S mice on day 11 PI. **(C)** Representative western immunoblot and **(D)** quantification of 15-Pgdh from colonic extracts of Se-D, Se-A, and Se-S mice on day 11 PI. **(E)** Shedding curve of *C. rodentium* infected Se-A mice treated with vehicle control or CAY10397. **(F)** Colon length of Se-A mice treated with control or CAY10397 on day 11 PI. **(G)** Colon length of Se-A mice treated with control or CAY10397 at day 30 PI. **(H)** Colonic crypt length of Se-A mice treated with control or CAY10397 on day 11 PI. Data indicate mean ± SEM of two independent experiments and n = 3-6 mice/group; one-way ANOVA **(D)** two-way ANOVA **(E)** unpaired *t*-test **(F–H)**. ^*^P < 0.05, ^**^P < 0.01, n.s., not significant.

Previous research in our laboratory has shown that Se supplementation of macrophages results in the shunting of the COX pathway of ARA metabolism from pro-inflammatory PGE_2_ to anti-inflammatory and pro-resolving 15d-PGJ_2_ (15). As in the DSS-colitis model reported earlier from our laboratory (17), we noticed an increasing trend in the expression of *Cox-2* (Ptgs2) in Se-D colonic tissue compared to the Se-A and Se-S mice following infection; however, the levels of 15-Pgdh, were significantly decreased on day 11 PI by 1.6 and 1.7 in Se-D colonic tissue when compared to Se-A and Se-S colonic tissues, respectively (Figures 3A–D). To further investigate the role of 15-Pgdh during infection, we used CAY10397, a well characterized inhibitor of 15-Pgdh activity (17, 36). Starting one day prior to infection, mice on Se-A diet were orally gavaged with 75 mg/kg of CAY10397 on alternate days until day 21 PI with *C. rodentium*. Interestingly, Se-A mice treated with CAY10397 experienced a greater bacterial burden from day 14 PI, that persisted until day 30 PI when compared to the vehicle control treated mice (Figure 3E). Furthermore, CAY10397 treated mice showed a 1.2-fold and 1.1-fold decrease in colon length at days 11 and 30, respectively, when compared to the control group (Figures 3F,G). We further observed an increase in the colonic crypt length of the CAY10397 treated mice compared to the control group (Figure 3H). Thus, treatment of Se-A mice with CAY10397 abrogated the protective effect of Se, suggesting that the oxidation of lipid mediators, including PGE_2_, is a key protective mechanism that is associated with Se levels, which needs to be examined in greater detail in future studies.

### Inhibition of 15-Pgdh Decreases the Expression of Zo-1

**Figure 4:**
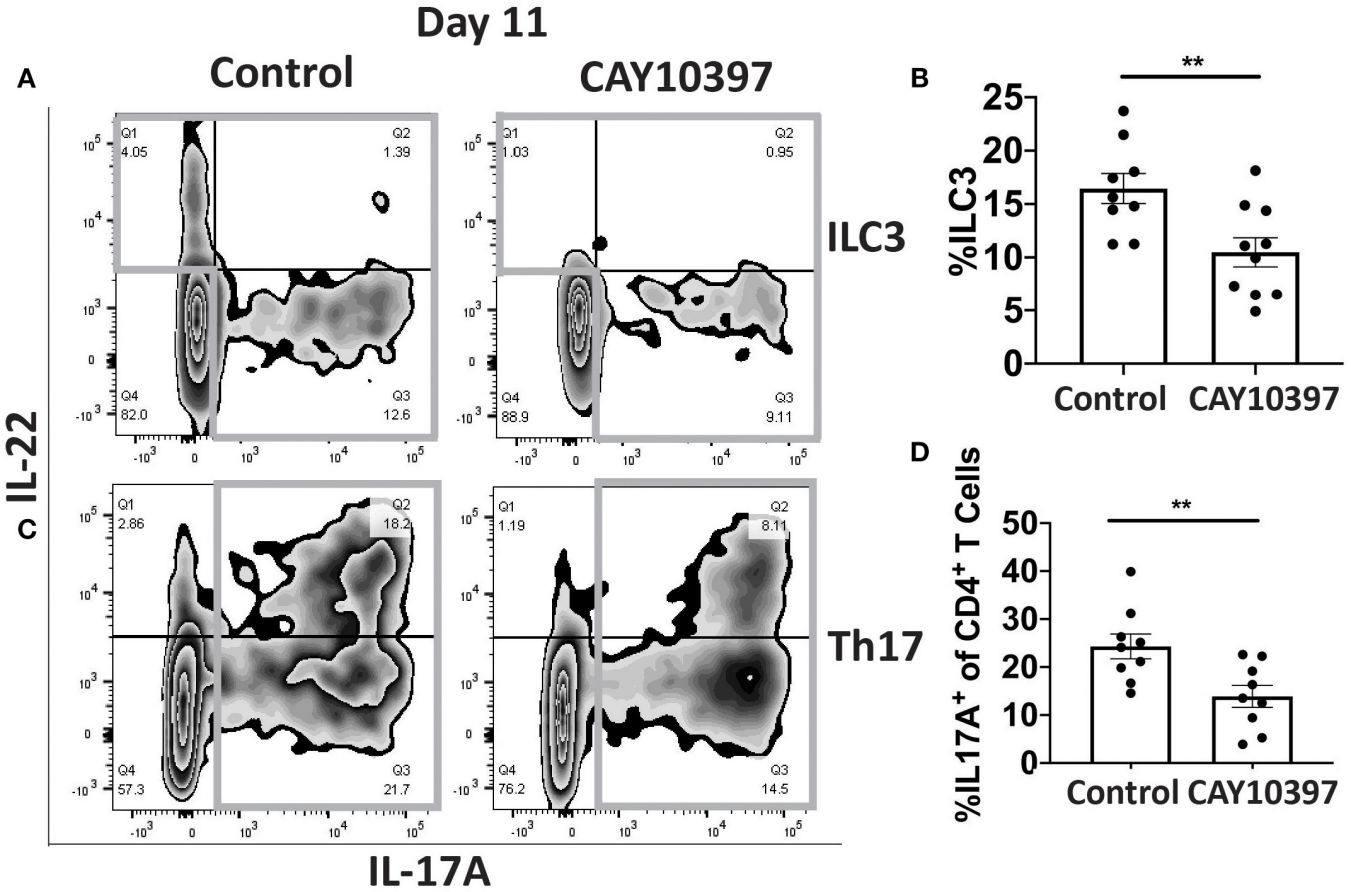
Inhibition of 15-Pgdh activity in Se-A mice impacts ILC3 and Th17 cells. **(A)** Representative flow plot and **(B)** quantification of ILC3s isolated from the cLP on day 11 PI. Gray box around representative flow plots indicate IL-17A^+^IL-22^+^ cells. **(C)** Representative flow plot and **(D)** quantification of IL-17A^+^ cells of CD4^+^ T cells indicating Th17 cells isolated from the cLP on day 11 PI. Gray box around representative flow plots indicate IL-17A^+^ cells. Data indicate mean ± SEM of two independent experiments and *n* = 9–10 mice/group; unpaired *t*-test **(B,D)** ^**^P < 0.01.

**Figure 5:**
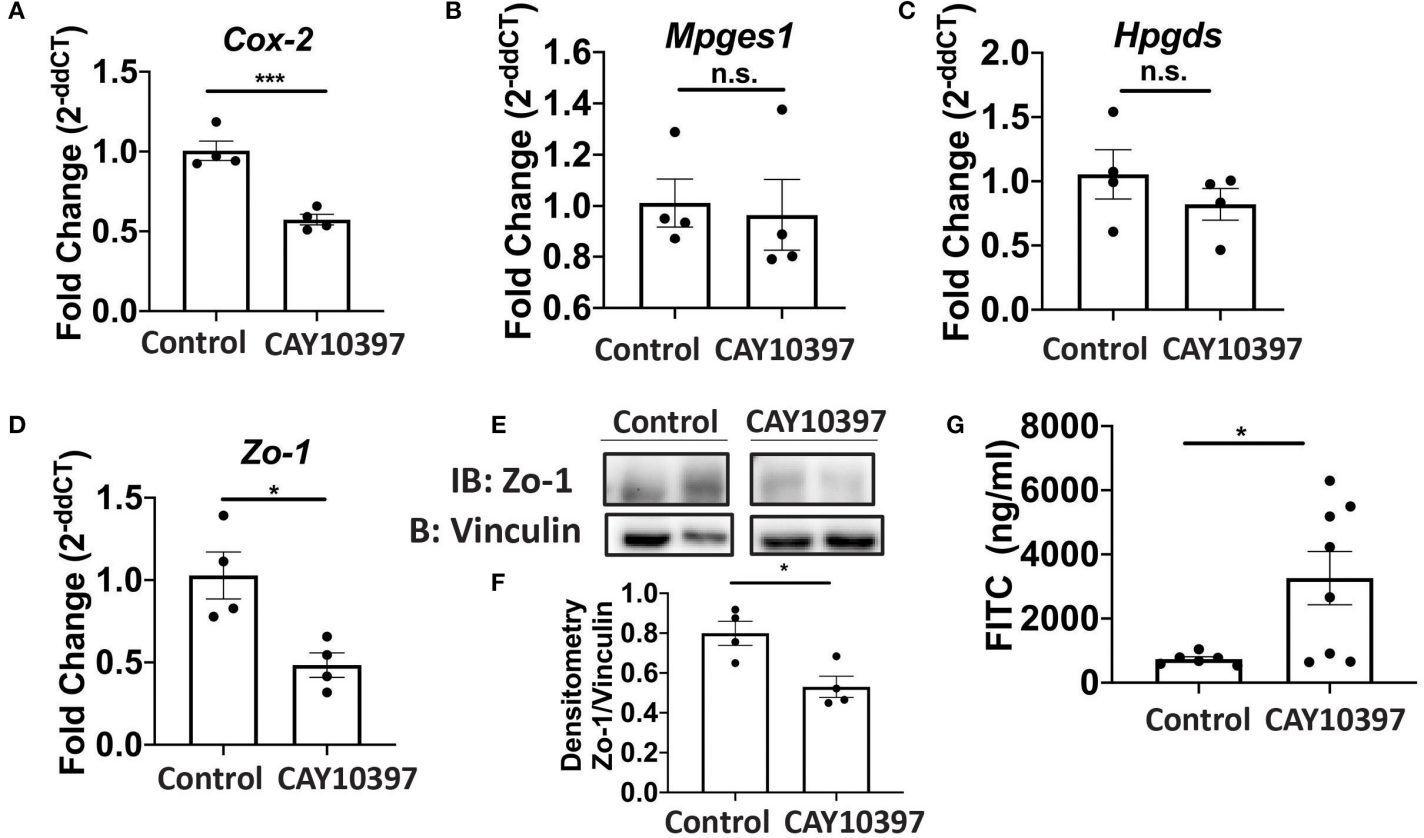
Activity of 15-Pgdh in Se-A mice is required for maintenance of the epithelial barrier integrity. mRNA expression of **(A)** *Cox2*, **(B)** *Mpges1*, **(C)** *Hpgds* and **(D)** *Zo-1* in the colon of Se-A mice treated with control or CAY10397 on day 11 PI. **(E)** Representative western immunoblot and **(F)** quantification of Zo-1 in the colonic extracts of Se-A mice treated with control or CAY10397 on day 11 PI. **(G)** FITC concentration in the serum of Se-A mice treated with control or CAY10397 on day 11 PI. Data indicate mean ± SEM of *n* = 4-8 mice/group, unpaired *t*-test **(A,D,F,G)** ^*^P < 0.05, ^***^P < 0.001, n.s., not significant.

We examined if 15-Pgdh activity in Se-A mice affected ILC3 and Th17 cells upon treatment with CAY10397. We observed a decrease in the percentage of ILC3s, as well as a decrease in the number of ILC3s per one million CD45^+^ cells in the CAY10397 treated mice when compared to the vehicle control (Figures 4A,B, Supplementary Figure 4A). A decrease in the percentage of Th17 cells, as well as a decrease in the number of Th17 cells per one million CD45^+^ cells in the CAY10397 treated mice were seen when compared to the vehicle control (Figures 4C,D, Supplementary Figure 4B). We also examined the humoral response in these mice by measuring serum anti- *C. rodentium* IgG and IgM and found that there were no differences between the CAY10937 treated mice and the control mice (Supplementary Figures 4C,D). Furthermore, CAY10397 decreased the expression of *Cox-2* by 0.4-fold compared to the vehicle control (Figure 5A). There was no difference in the expression of *Mpges1*, the enzyme that leads to the production of PGE_2_, while a trend in decrease in the expression of *Hpgds*, the enzyme that results in the production of PGD_2_, was observed (Figures 5B,C). Given that PGE_2_ is a potent proangiogenic factor, the effect of inhibition of PGE_2_ oxidation on the epithelial barrier was examined using the expression of *Zo-1* as a surrogate marker. CAY10397 treatment resulted in a 0.5-fold decrease in the expression of *Zo-1* on day 11 PI at the mRNA level (Figure 5D). Further analysis using western immunoblotting and IHC indicated a decrease in Zo-1 expression in the CAY10397 treated group, which corroborated with the increase in systemic levels of FITC- dextran of these mice following oral gavage (Figures 5E–G, Supplementary Figure 5). In summary, these results suggest that the increased expression of 15-Pgdh under Se replete conditions potentially plays an important role in maintaining the epithelial barrier integrity.

### Administration of 15d-PGJ_2_ Increases ILC3s, Th17 Cells, and Zo-1 Expression

**Figure 6:**
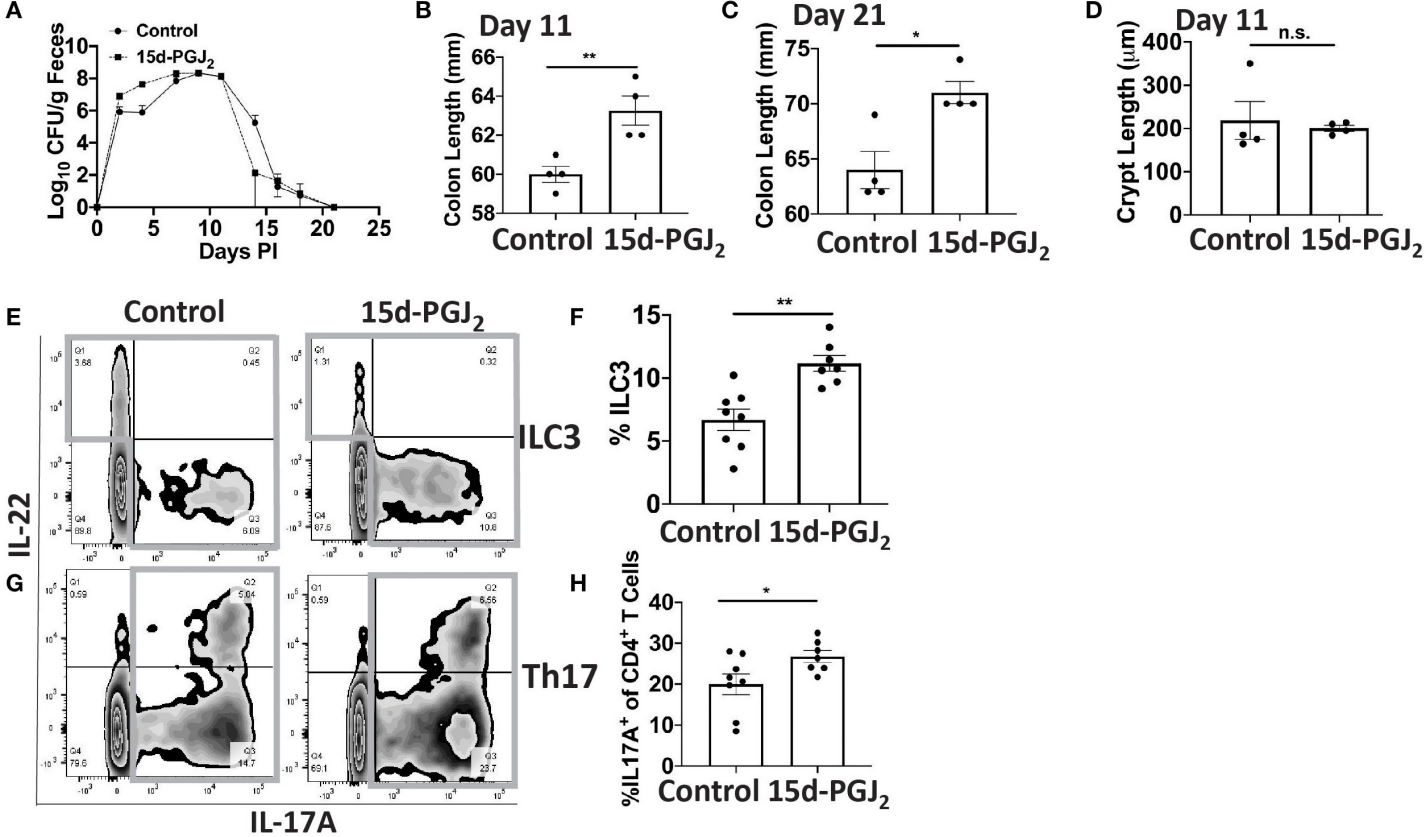
Treatment of Se-D mice with 15d-PGJ_2_ increases ILC3 and Th17 cells PI. Se-D mice were treated with 15d-PGJ_2_ at 0.05 mg/kg intraperitoneally on day −1 PI followed by daily injections. **(A)** Shedding curve of *C. rodentium* infected Se-D mice treated with control or 15d-PGJ_2_. **(B)** Colon length of Se-D mice treated with control or 15d-PGJ_2_ on day 11 PI. **(C)** Colon length of Se-D mice treated with control or 15d-PGJ_2_ on day 21 PI. **(D)** Colonic crypt length of Se-D mice treated with control or 15d-PGJ_2_ on day 11 PI. **(E)** Representative flow plot and **(F)** quantification of ILC3s isolated from the cLP on day 11 PI. Gray box around representative flow plots indicate IL-17A^+^IL-22^+^ cells. **(G)** Representative flow plot and **(H)** quantification of IL-17A^+^ cells of CD4^+^ T cells indicating Th17 cells isolated from the cLP on day 11 PI. Gray box around representative flow plots indicate IL-17A^+^ cells. Data indicate mean ± SEM of two independent experiments and n = 4–8 mice/group, unpaired *t*-test **(B,C,E,G)** ^*^P < 0.05, ^**^P < 0.01, n.s., not significant.

**Figure 7:**
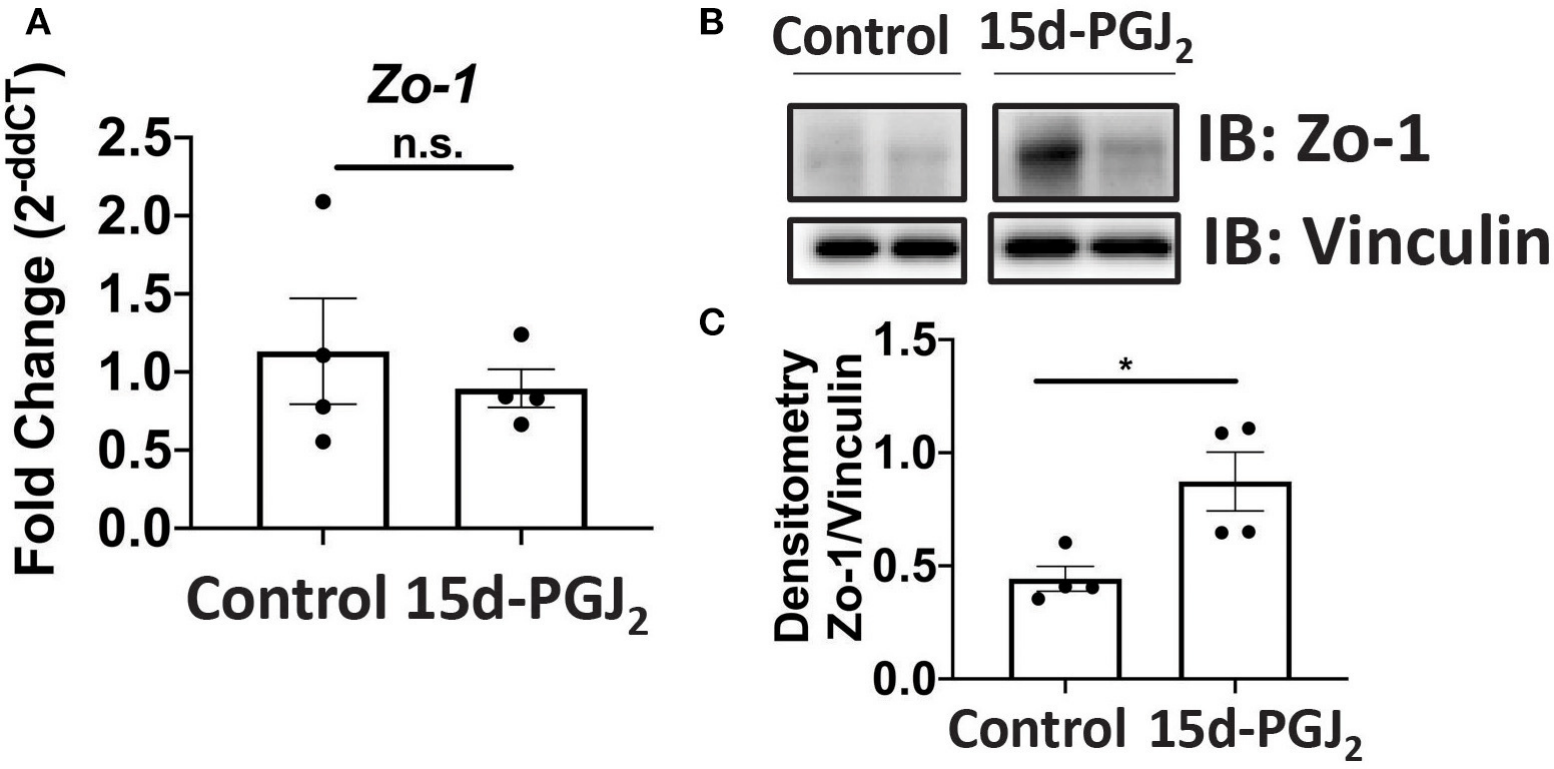
15d-PGJ_2_ treatment of Se-D mice increases Zo-1 in Se-D mice PI. **(A)** *Zo-1* mRNA expression in colon of Se-D mice treated with control or 15d-PGJ_2_ on day 11 PI. **(B)** Representative western immunoblot and **(C)** quantification of Zo-1 protein in the colonic extracts of Se-D mice treated with control or 15d-PGJ_2_ on day 11 PI. Data indicate mean ± SEM of *n* = 4 mice/group, unpaired *t*-test **(C)** *P < 0.05, n.s., not significant.

Given that Se-dependent anti-inflammatory effects were mediated, in part, by 15d-PGJ_2_ (15, 16), we examined the effect of 15d-PGJ_2_ during infection. Se-D mice were administered 0.05 mg/kg 15d-PGJ_2_ one day prior to infection, via intraperitoneal injection, and continued daily until day 18 PI. Compared to the PBS (sham) treated mice, we observed a decreased trend in the bacterial burden at day 14 PI in the 15d-PGJ_2_ treated group (Figure 6A). The 15d-PGJ_2_ treated mice, despite being on the Se-D diet, also showed longer colons compared to the vehicle control mice at day 11 PI, which persisted even at day 21 PI when the mice cleared the infection (Figures 6B,C). Surprisingly, we observed no difference in the colonic crypt length between the 15d-PGJ_2_ treated and the vehicle control mice at day 11 PI (Figure 6D). Interestingly, we observed an increase in the percentage of ILC3s, but not in the number of ILC3s per one million CD45^+^ cells in the 15d-PGJ_2_ treated mice (Figures 6E,F, Supplementary Figure 6A). Additionally, we observed an increase in the percentage of Th17 cells as well as an increase in the number of Th17 cells per one million CD45^+^ cells in the 15d-PGJ_2_ treated mice (Figures 6G,H, Supplementary Figure 6B). Furthermore, no differences were seen in serum levels of anti- *C. rodentium* IgG and IgM between the 15d-PGJ_2_ and vehicle treated mice (Supplementary Figures 6C,D). To further examine the role of 15d-PGJ_2_ on the epithelial barrier integrity, the colons of both control and 15d-PGJ_2_ treated mice were subjected to qPCR, western blot, and IHC for Zo-1 expression. While there were no observed differences in the mRNA, western immunoblot analysis revealed a 2-fold increase in the expression of Zo-1 in the 15d-PGJ_2_ treated mice on Se-D diet compared to the control mice on Se-D diet (Figures 7A–C). Similarly, there was an increase in the expression of Zo-1 demonstrated by IHC (Supplementary Figure 7) suggesting that 15d-PGJ_2_ may play a role in mediating the Se-dependent protection of epithelial barrier integrity via post-transcriptional or translational mechanisms that need to be further elucidated.

### Selenoproteins in Macrophages Are Crucial for Survival From Infection

**Figure 8:**
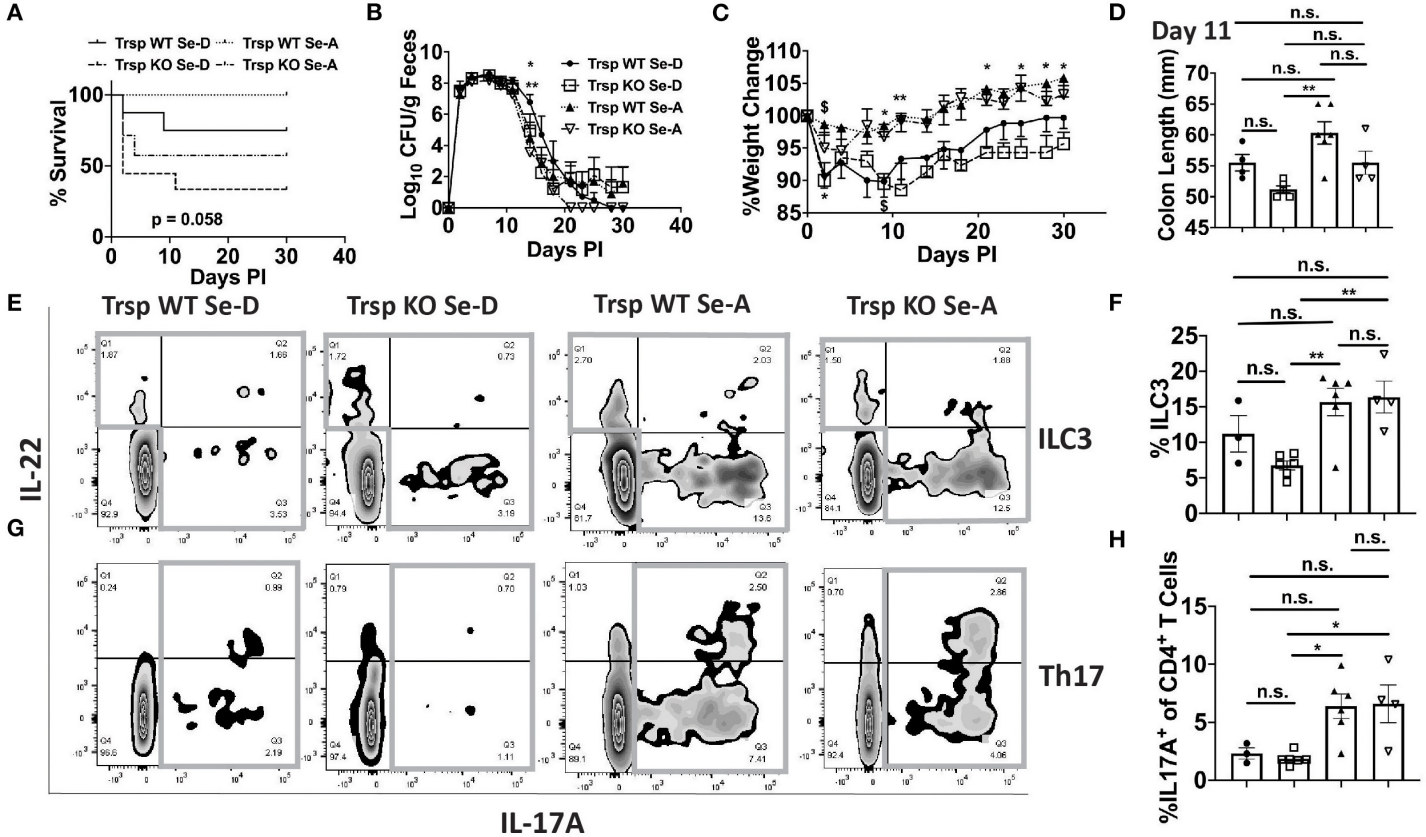
Lack of selenoprotein expression in macrophages decreases survival following infection. **(A)** Survival curve of T*rsp*^*fl*/*fl*^ WT and *Trsp*^*fl*/*fl*^ *LysM*^*Cre*^ (*Trsp* KO) mice on Se-D and Se-A diets. **(B)** Shedding curve of *Trsp*^*fl*/*fl*^ WT and *Trsp* KO mice on both Se-D and Se-A diet. (*comparing Trsp WT on Se-D vs. Trsp WT on Se-A at day 14; **comparing Trsp WT Se-D vs. Trsp KO Se-A at day 14) **(C)** Percent weight change of *Trsp* WT and *Trsp* KO mice on Se-D and Se-A diet. $ represents *comparing Trsp WT on Se-D vs. Trsp WT on Se-A on days 2, 9 PI; *comparing Trsp KO on Se-D vs. Trsp WT on Se-A on days 2, 9, 21, 25, 28, and 30 PI; **comparing Trsp KO on Se-D vs. Trsp WT on Se-A on day 11 PI. **(D)** Colon length of *Trsp* WT and *Trsp* KO mice on Se-D and Se-A diet on day 11 PI. **(E)** Representative flow plot and **(F)** quantification of ILC3s isolated from the cLP on day 11 PI. Gray box around representative flow plots indicate IL-17A^+^IL-22^+^ cells. **(G)** Representative flow plot and **(H)** quantification of IL-17A^+^ cells of CD4^+^ T cells indicating Th17 cells isolated from the cLP on day 11 PI. Gray box around representative flow plots indicate IL-17A^+^ cells. Data indicate mean ± SEM of two independent experiments and *n* = 3-9 mice/group, two-way ANOVA **(B,C)**, one-way ANOVA **(D,F,H)**. *P < 0.05, **P < 0.01, n.s., not significant.

Given that macrophages are one of the major producers of PGE_2_ and 15d-PGJ_2_ (15) and also serve as innate immune cells that play a central role in combatting enteric microbial infections (37), we examined whether selenoproteins in macrophages were a critical component in combating the infection. The bacterial shedding curve, weight change, and survival of *Trsp*^*fl*/*fl*^*LysM*^*Cre*^ (*Trsp* KO) mice, which lack the ability to express selenoproteins in monocytes and macrophages, and their controls (*Trsp*^*fl*/*fl*^; *Trsp* WT) were examined for 30 days PI. *Trsp* KO and WT mice on Se-D diet, as well as *Trsp* KO mice on Se-A diet experienced a higher mortality rate when compared to the *Trsp* WT mice on Se-A diet, with the *Trsp* KO mice on Se-D diet exhibiting the highest mortality of 66% (Figure 8A). A significant difference in the shedding curve was seen on day 14 PI between *Trsp* WT Se-A and Se-D mice (Figure 8B). *Trsp* WT and KO mice on Se-D diet also experienced greater percent weight change when compared to the *Trsp* WT on Se-A diet (Figure 8C). The *Trsp* KO mice on Se-D diet experienced shorter colons when compared to the *Trsp* WT on Se-A diet, while there was also a trend observed in shorter colons when comparing *Trsp* WT mice on Se-D diet and *Trsp* KO mice on Se-A diet to *Trsp* WT mice on Se-A diet (Figure 8D). Furthermore, we also characterized the role of monocyte/macrophage selenoproteins in the differentiation of colonic ILC3 and Th17 cells. Surprisingly, we observed a higher percentage of ILC3s in both *Trsp* WT and KO mice on Se-A diet compared to *Trsp* KO on Se-D diet, and a trend in the increase in the percentage of ILC3s when comparing *Trsp* WT mice on Se-A diet vs. *Trsp* WT mice on Se-D diet (Figures 8E,F). These differences and trends were also observed in the number of ILC3s per one million CD45^+^ cells (Supplementary Figure 8A). The *Trsp* WT and KO mice on Se-A diet also showed an increase in the percentage of Th17 cells when compared to *Trsp* KO mice on Se-D diet, while there was a trend in the increase of the percentage of Th17 cells when compared to Trsp WT mice on Se-D diet (Figures 8G,H). Again, the same trends and differences were observed in the number of Th17 cells per one million CD45^+^ cells. No differences in the serum levels of anti-*C. rodentium* IgG and IgM were observed at day 11 PI (Supplementary Figures 8C,D). These results suggest that lack of selenoprotein expression in macrophages is crucial for survival of *C. rodentium* but not through their impact on ILC3s and Th17 cells.

## Discussion

The global burden of enteric infections continues to be of great concern on both health and economic fronts (38). While the current treatment for enteric infections has resulted in a decline in infant mortality, preventative measures still need to be taken (38). Furthermore, while enteric infections can impact nutritional status (38), it is clear that nutritional deficiencies can also have a bearing on infections with regard to progression and timely resolution. Here, we focused on how nutritional deficiency in Se impacts enteric infections in a model of *C. rodentium* and the role of Se in modulating ILC3 and Th17 differentiation and epithelial barrier integrity to promote efficient resolution.

While studies of injury to the GI tract have focused on the role of Se in certain bacterial and viral infections as well as preclinical models of IBD, there are not many studies that report on the effect of Se during bacterial infections, specifically enteric infections that cause GI inflammation (17, 29, 39, 40). A previous study suggested Se in conjunction with vitamin E to be effective in reducing the *C. rodentium* burden in mice (29). Here we further extended the studies to address the role of Se on the immune mechanisms in the gut LP that also help in maintaining the integrity of the epithelial barrier during an enteric infection.

The notion that Se serves in a protective role during enteric infection is supported by the fact that Se-D mice succumbed to infection following administration of *C. rodentium* and that these mice had lower ILC3s and Th17 cells, which are required for clearance of the bacteria. Preclinical and clinical studies have demonstrated that Se deficiency negatively impacted the host's ability to mount an immune response required to combat infections (41). In addition, deletion of selenoproteins in T-cells resulted in the inability of these cells to mature and be activated (42). Our current studies provide further evidence that indeed Se is crucial for mounting an appropriate immune response. Therefore, the lower percentage of Th17 cells may be due to a decrease in T cell differentiation or maturation that needs to be further elucidated. Furthermore, the fact that we observed no statistical differences in Th17 cells when comparing Se-D and Se-A mice, but observed an increase in the ILC3s in the Se-A mice compared to the Se-D mice, suggests that ILC3s may be responsible for the protective response observed in these mice. The humoral response is also an integral component to the clearance of the bacteria (35). However, we observed no differences in IgG and IgM in the sera of these mice, which may be attributed to the time point of analysis. Previous reports have shown that the response increases with time, where the immunoglobulin levels are greater at days 15 and 21 PI (35). Further studies are needed to clarify the role of humoral immune mechanisms in the context of Se and enteric bacterial infections, where these antibody responses should be examined at later time points (day 15 PI and beyond) followed by reinfection studies. Interestingly, we observed an increase in the expression of Zo-1 in response to dietary Se. To our knowledge, this is the first ever report to causally link the expression of Zo-1 to dietary Se status during an enteric infection. These findings also suggest that selenoproteins may be involved in maintaining the epithelial barrier integrity by influencing the expression of barrier proteins. Further studies are currently in progress to identify the underlying molecular mechanisms.

Data showing higher expression of 15-Pgdh in the colonic tissue of mice replete with Se corroborated with our previous studies in a chemical injury model of colitis (17). However, in contrast to our previous report where DSS was used to elicit inflammation, treatment of mice with CAY10397 resulted in a decrease in *Cox2* mRNA. This paradoxical finding may be a result of the time point chosen and/or the type of inflammatory agent used. It is possible that Cox2 is temporally regulated and may be increased at earlier or later days PI, thereby playing a dual role in inflammation and also during resolution. Furthermore, arachidonic acid could still be metabolized through Cox1, which could increase PGE_2_ in CAY10397 treated mice PI. More studies are needed to delineate the role of the two enzymes in the context of enteric infections. Regardless, pharmacological inhibition of 15-Pgdh with CAY10397 indicates its importance in differentiation of Th17 and ILC3 cells. Interestingly, our studies also indicate 15-Pgdh to be crucial for the maintenance of the epithelial barrier integrity, which is evident by the decrease in Th17 and an increase in intestinal permeability as demonstrated by a functional assay with FITC dextran. Such a causal association of 15-Pgdh expression with dietary Se levels strongly suggests a key role for this enzyme in the oxidation of bioactive lipid mediators that are both beneficial and detrimental. PGE_2_, though important in the maintenance of the epithelial barrier integrity, also serves as a pro-angiogenic signal during inflammation to initiate early steps in tumorigenesis (43, 44). We believe that oxidation of PGE_2_ to its downstream metabolites, 15-keto-PGE_2_ and 13,14 dihydro-15-keto-PGA_2_, may serve as an important mediator in resolving inflammation. In this regard, our previous studies demonstrate that Se increases PGE_2_ oxidation products, while increasing the endogenous levels of PGD_2_ and its metabolites (17, 45). Moreover, PGD_2_ metabolites, Δ^12^-PGJ_2_ and 15d-PGJ_2_, and PGE_2_ oxidation product, 13,14 dihydro-15-keto-PGA_2_, share the same cyclopentenone structure, suggesting a potential overlap in mechanisms underlying alleviation of inflammation. However, the likelihood of achieving such concentrations in colonic tissues to locally impact pathways of inflammation and resolution are unclear and will need to be examined.

Alleviation of the effects of *C. rodentium* infection by 15d-PGJ_2_, seen in the form of increased colon length and higher expression of Th17 and ILC3 cells along with increased expression of Zo-1, further support our previous findings that selenoproteins exert their effects through the shunting of the ARA pathway from pro-inflammatory mediators to anti-inflammatory and pro-resolving mediators. Potential mechanisms include activation of PPARγ by endogenous cyclopentenone prostaglandins (such as 15d-PGJ_2_), which is highly expressed in the epithelial cells of the colon (in addition to adipocytes) (46). In support, preliminary studies suggest an increase in PPARγ target genes, such as *Cd36* in the 15d-PGJ_2_ treated mice infected with *C. rodentium* (data not shown). Biopsies from ulcerative colitis patients with gut inflammation showed decreased expression of PPARγ in their inflamed colon (47). Furthermore, administration of rosiglitazone, a synthetic PPARγ agonist, attenuated the colonic damage by increasing the expression of epithelial barrier protein following induction of murine colitis with DSS (48). Since chronic enteric infections can lead to IBD, our studies further support the importance of finding alternative methods to negatively impact such a progression leading to IBD. Interestingly, in line with previous studies from our laboratory, selenoproteins in macrophages appear to be an integral part of the mechanism of action, since macrophages are one of the major local producers of these endogenous bioactive lipids that modulate diverse pathways, including PPARγ and NF-κB (15, 16). Studies using the *Trsp*^*fl*/*fl*^*LysM*^*Cre*^ mice further confirmed their importance, given that addition of Se to these mice failed to prevent the lethal effect of infection. The high mortality rate of these mice may be attributed to a defective recruitment or activation of innate immune cells, such as monocytes, macrophages, dendritic cells, and other granulocytes, which are crucial for combatting the bacteria during the early stages of infection. Further studies are currently being planned to investigate this. While we observed a higher percentage of ILC3s and Th17 cells in the Trsp^*fl*/*fl*^ mice on Se-A diet when compared to the *Trsp*^*fl*/*fl*^ *LysM*^*Cre*^ mice on Se-D, we surprisingly did not observe any difference when the mice were compared to the *Trsp*^*fl*/*fl*^ on Se-D diet and *Trsp*^*fl*/*fl*^ *LysM*^*Cre*^ mice on Se-A diet. These findings suggest selenoproteins in other immune cell types such as neutrophils and dendritic cells may compensate for the lack of selenoproteins in the macrophages. These findings also suggest that elemental Se may also play a role in the compensation of lack of selenoproteins in macrophages, since the Trsp^*fl*/*fl*^ mice and *Trsp*^*fl*/*fl*^ *LysM*^*Cre*^ mice on Se-A diet had comparable percentages of ILC3s and Th17 cells.

In conclusion, our studies demonstrate that dietary Se, through the action of selenoproteins and endogenous bioactive lipid meditators derived from PGD_2_, plays a protective role in enteric infections by regulating ILC3 and Th17 cells needed to efficiently resolve infection and maintain the gut epithelial barrier. Our data suggest that dietary supplementation with Se could be exploited as a potential adjuvant therapy to effectively resolve inflammation as seen during enteric infections and other associated diseases such as IBD.

## Data Availability Statement

The datasets generated for this study are available on request to the corresponding author.

## Ethics Statement

The animal study was reviewed and approved by Institutional Animal Care and Use Committee.

## Author Contributions

SN and KP conceived the project, designed research, analyzed data, and wrote the paper. SN, LZ, FQ, MH, and BA performed research. BC developed *Trsp* KO mice and contributed to the manuscript preparation. VS and NX provided expertise with IHC and flow cytometry, respectively. DD and SA synthesized CAY10397 and provided input on the manuscript. All authors contributed to the article and approved the submitted version.

## Conflict of Interest

The authors declare that the research was conducted in the absence of any commercial or financial relationships that could be construed as a potential conflict of interest.
