# Supplementary material for: The Essential Role of Selenoproteins in the Resolution of Citrobacter rodentium-Induced Intestinal Inflammation
Source: Front Nutr. 2020 Jul 8;7:96. doi: 10.3389/fnut.2020.00096 (PMC7381334; doi:10.3389/fnut.2020.00096)
Supplement: Supplementary file 1 [file Data_Sheet_1.docx]

***Supplementary Material***

**
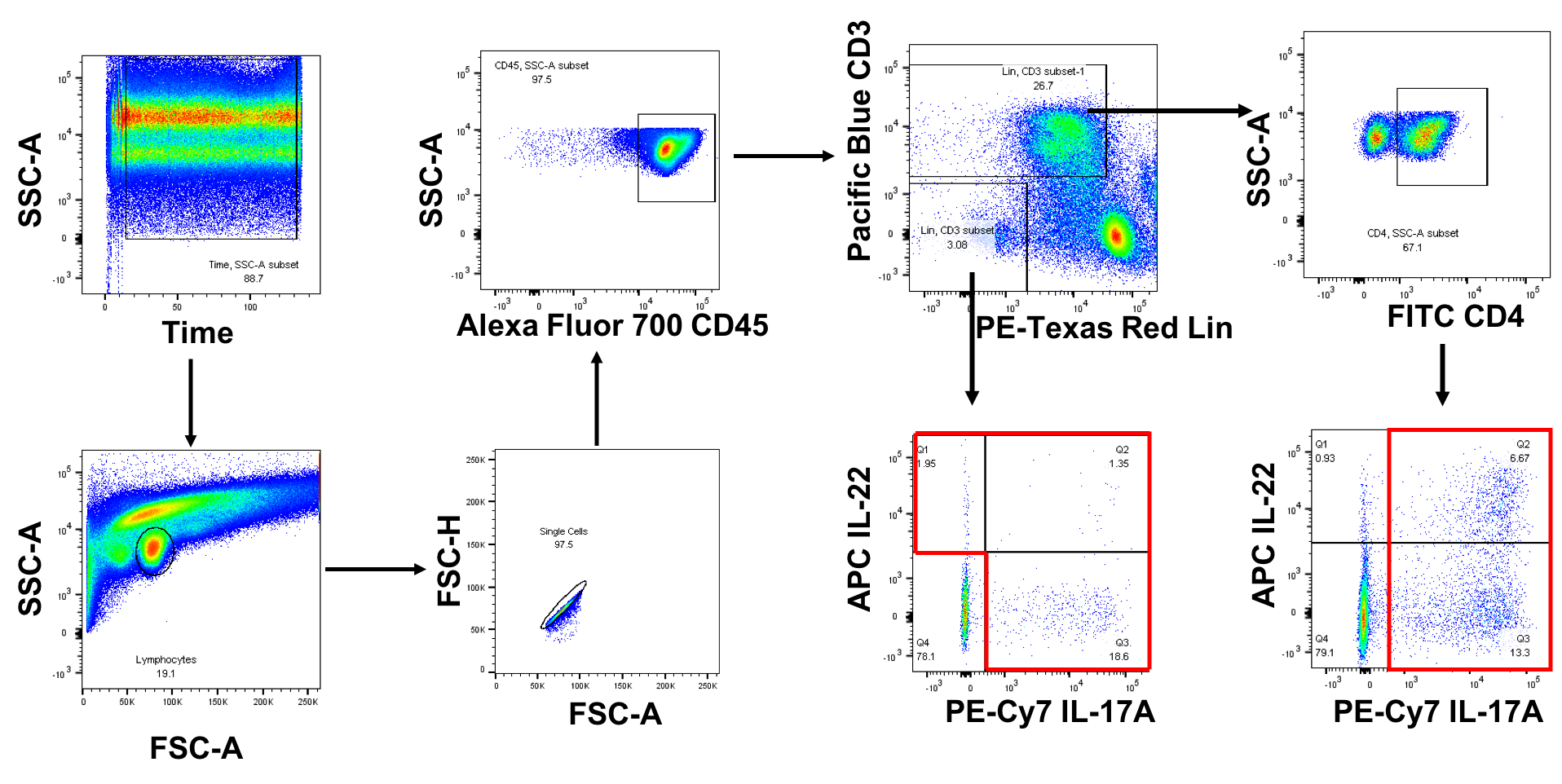
**

**Supplementary Fig 1. Schematic representation showing flow cytometry gating strategy.** Gating strategy used to delineate immune cells isolated from the colonic LP into ILC3s: CD45^+^Lin^-^CD3^-^IL-17A^+^IL-22^+^ and Th17 cells: CD45^+^CD3^+^CD4^+^IL-17A^+^.


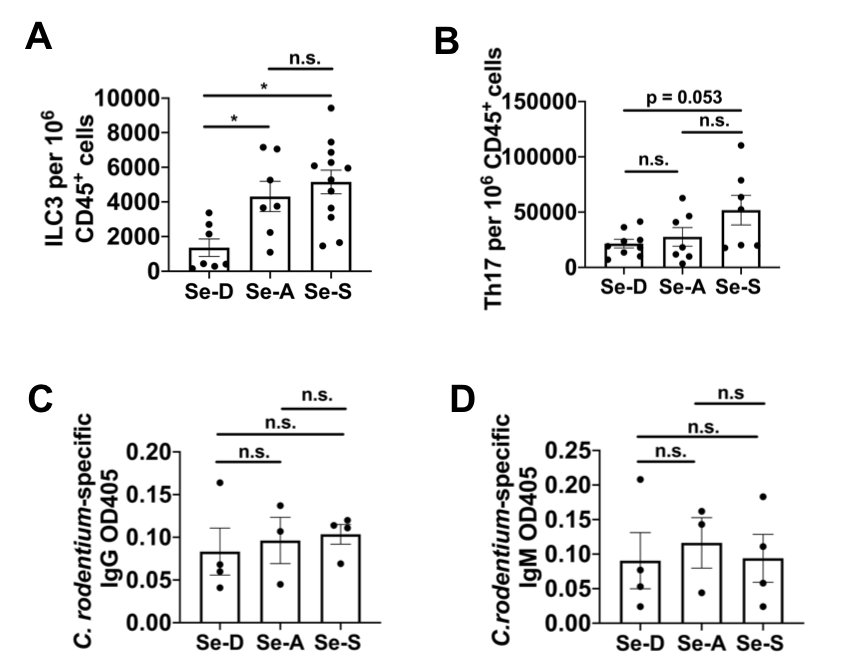


**Supplementary Fig 2. ILC3 and Th17 cell numbers and serum anti *C. rodentium* IgG and IgM.** C57BL/6 mice were maintained on Se-D, Se-A, or Se-S diets for eight weeks followed by infection with *C. rodentium* via oral gavage. **a, b** ILC3s and Th17 cells per one million CD45^+^ cells isolated from the cLP at day 11 PI. **c, d** serum anti-*C. rodentium* IgG and IgM. Data indicate mean $\pm$ SEM of two to three independent experiments and n = 4-12nmice/group; one-way ANOVA **(a,b,c,d)** *P<0.05, n.s., not significant.

**
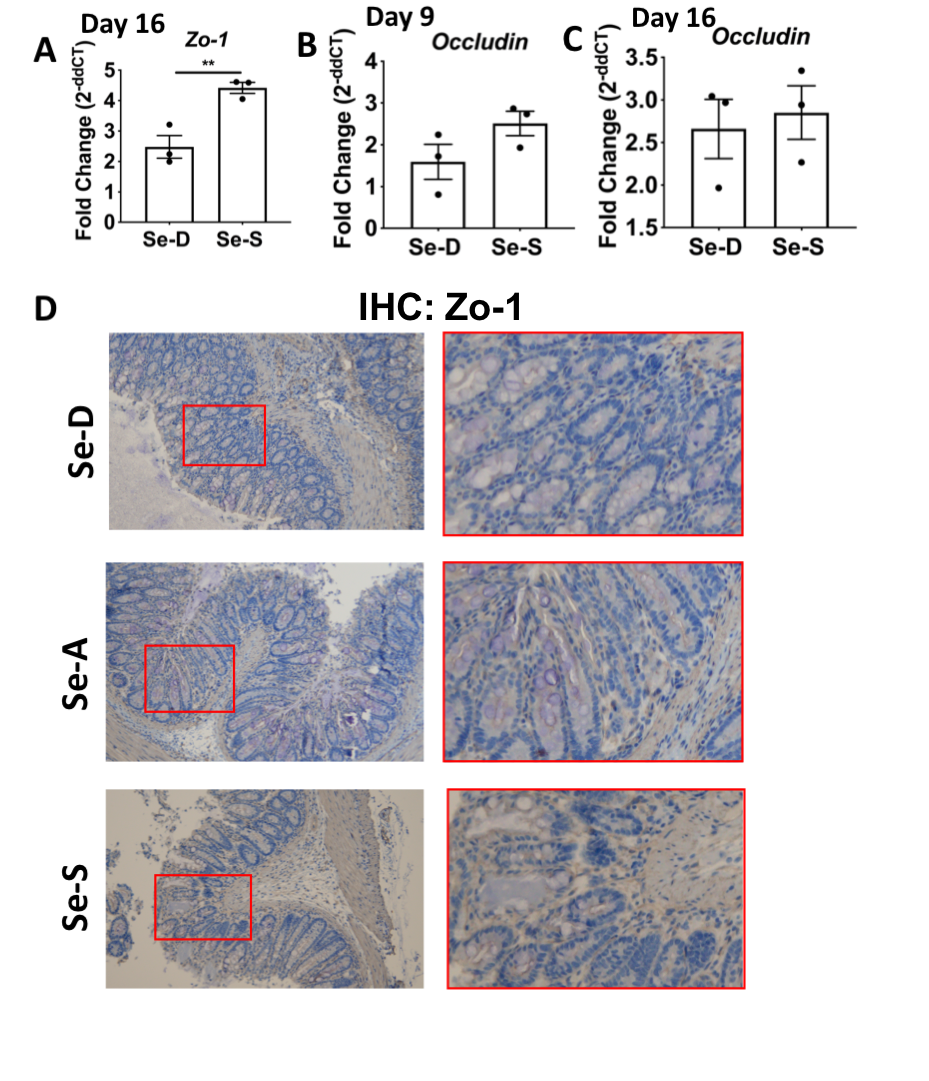
**

**Supplementary Fig 3. Zo-1 and occludin expression in colon tissue.** C57BL/6 mice were maintained on Se-D, Se-A, or Se-S diets for eight weeks followed by infection with *C. rodentium* via oral gavage. **a** *Zo-1* mRNA expression in colon of mice on Se-D and Se-S diets at day 16 PI. **b, c** *Occludin* mRNA expression in colon of mice on Se-D and Se-S diets at days 9 and 16 PI. **d** Representative images of IHC staining with Zo-1(brown staining) in the distal colons of the above mice on day 11 PI. Data indicate mean $\pm$ SEM of n= 3 mice/group, unpaired t-test **(a)** *P<0.05


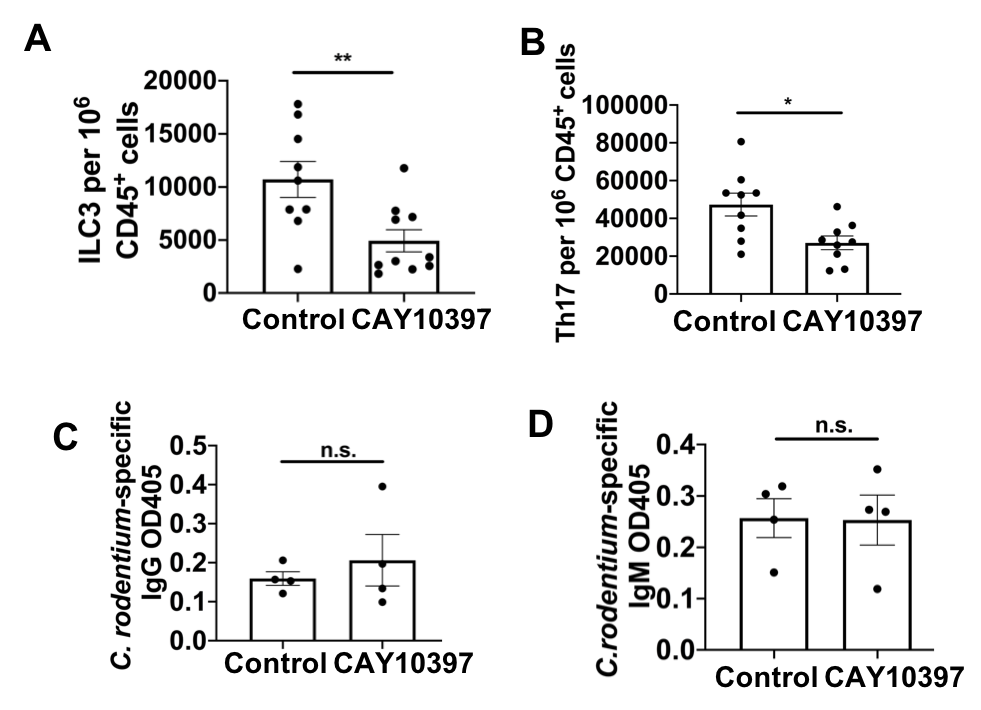


**Supplementary Fig 4. ILC3 and Th17 cell numbers and serum anti *C. rodentium* IgG and IgM**. Mice maintained on Se-A diet were treated with CAY10397 in PBS containing DMSO and sodium carbonate at 75 mg/kg by oral gavage a day before infection followed by administration every alternate day post infection until day 21 PI. Sterile PBS containing DMSO and sodium carbonate was used as the control arm for comparison. **a, b** ILC3s and Th17cells per one million CD45^+^ cells isolated from the cLP at day 11 PI. **c, d** serum anti-*C. rodentium* IgG and IgM. Data indicate mean $\pm$ SEM of two independent experiments, n = 4-10 mice/group; unpaired t-test **(a,b,c,d)** *P<0.05, **P<0.01, n.s., not significant

**Supplementary Fig 5.** **Zo-1 expression in colon of CAY10397 treated mice.** Mice maintained on Se-A diet were treated with CAY10397 in PBS containing DMSO and sodium carbonate at 75 mg/kg by oral gavage a day before infection followed by administration every alternate day post infection until day 21 PI. Sterile PBS containing DMSO and sodium carbonate was used as the control arm for comparison. Representative images of IHC staining with Zo-1 (brown staining) in the distal colons of Se-A mice treated with control or CAY10397 on day 11 PI.

**
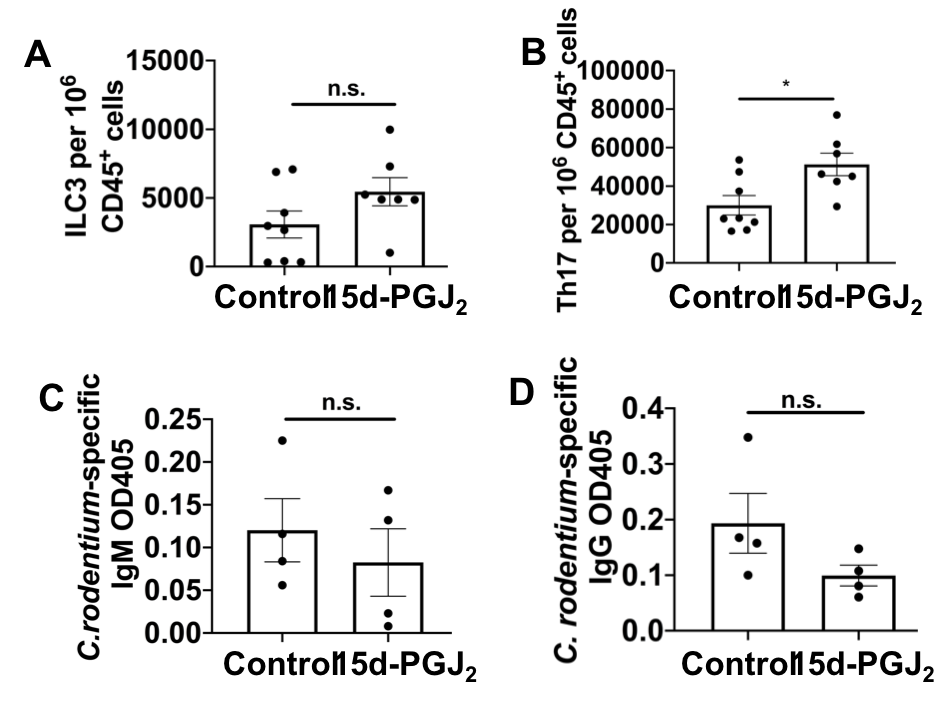
**

**Supplementary Fig 6. ILC3 and Th17 cell numbers and serum anti *C. rodentium* IgG and IgM.** Mice maintained on Se-D diet were treated with 15d-PGJ_2_ in PBS at 0.05 mg/kg intraperitoneally a day before infection followed by daily injections post infection. Sterile PBS-treatment was used as the control arm for comparison. **a, b** ILC3s and Th17 cells per one million CD45^+^ cells isolated from the cLP at day 11 PI. **c, d** serum anti-*C. rodentium* IgG and IgM. Data indicate mean $\pm$ SEM of two independent experiments and n = 4-8 mice/group; unpaired t-test **(a,b,c,d)** *P<0.05, n.s., not significant.

**
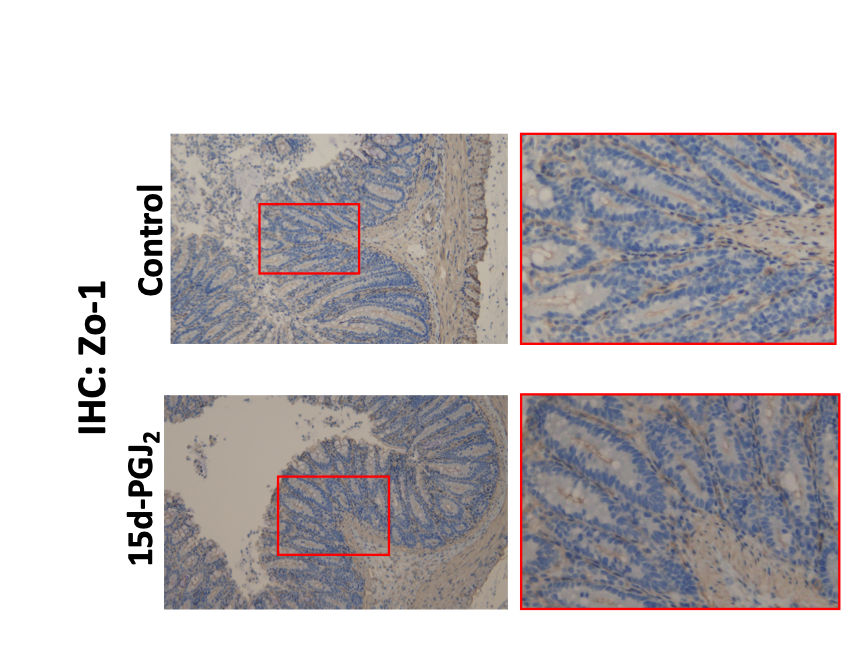
**

**Supplementary Fig 7.** **Zo-1 expression in colons of 15d-PGJ_2_ treated mice**. Mice maintained on Se-D diet were treated with 15d-PGJ_2_ in PBS at 0.05 mg/kg intraperitoneally a day before infection followed by daily injections post infection. Sterile PBS-treatment was used as the control arm for comparison. Representative images of IHC staining with Zo-1 (brown staining) in the distal colons of Se-D mice treated with control or 15d-PGJ_2_ on day 11 PI.

**
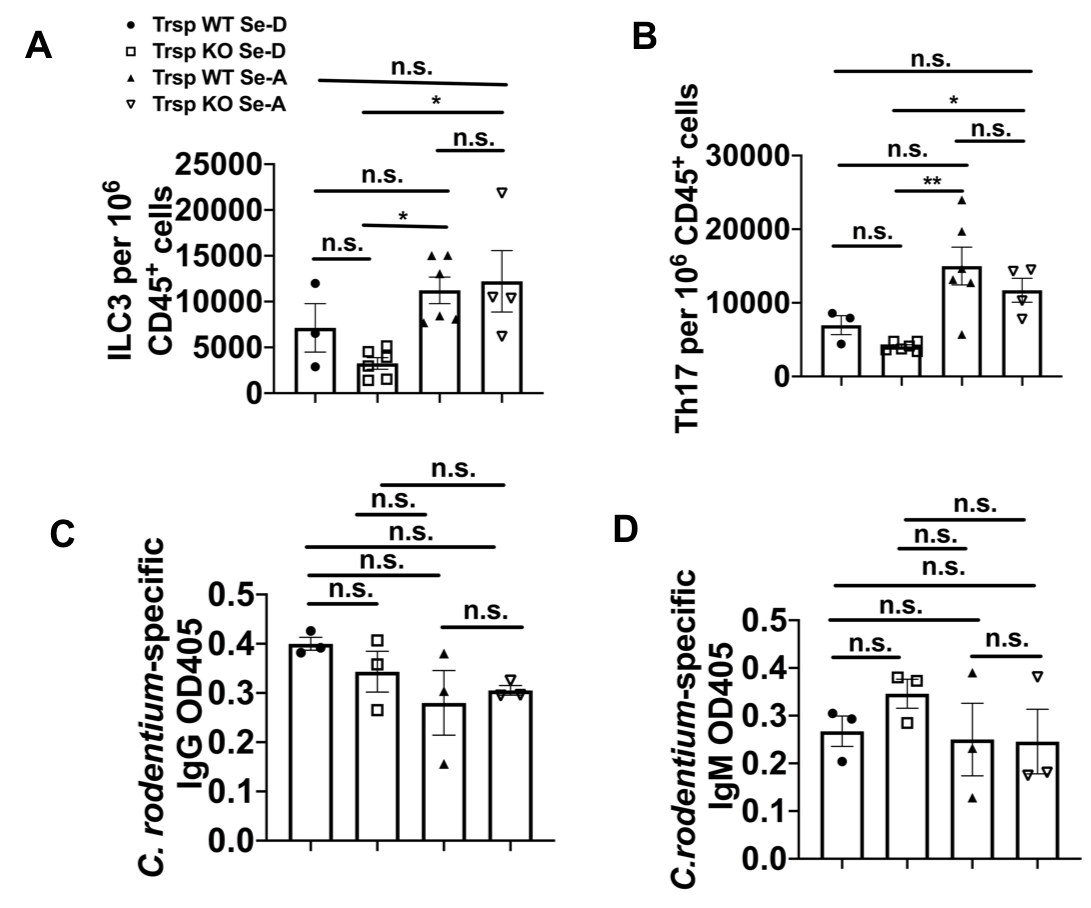
**

**Supplementary Fig 8. ILC3 and Th17 cell numbers and serum anti *C. rodentium* IgG and IgM.** Trsp WT and KO mice were maintained on Se-D or Se-A diets for eight weeks followed by infection with *C.rodentium* via oral gavage. **a, b** ILC3s and Th17 cells per one million CD45^+^ cells isolated from the cLP at day 11 PI. **c, d** serum anti-*C. rodentium* IgG and IgM. Data indicate mean $\pm$ SEM of two independent experiments and n = 3-6 mice/group; one-way ANOVA **(a,b,c,d)** *P<0.05, **P<0.01 n.s., not significant.
